# Study on Deterioration of Gray Brick with Different Moisture Contents under Freeze–Thaw Environment

**DOI:** 10.3390/ma15051819

**Published:** 2022-02-28

**Authors:** Jianwei Yue, Can Ma, Limin Zhao, Qingmei Kong, Xiangchun Xu, Zifa Wang, Ying Chen

**Affiliations:** 1 School of Civil Engineering and Architecture, Henan University, Kaifeng 475004, China; yjw@vip.henu.edu.cn (J.Y.); mc0524618@163.com (C.M.); 10160004@vip.henu.edu.cn (Q.K.); zf_wang@henu.edu.cn (Z.W.); 104754190856@henu.edu.cn (Y.C.); 2 Key Laboratory for Restoration and Safety Evaluation of Immovable Cultural Relics in Kaifeng City, Kaifeng 475004, China; 3 Yellow River Civilization and Sustainable Development Research Center, Henan University, Kaifeng 475004, China

**Keywords:** gray brick, moisture content, freeze–thaw cycle, mechanical property, freeze–thaw damage

## Abstract

Generally, brick buildings are in the open-air environment year round, and damage to them is aggravated by the effect of repeated freezing and thawing cycles. In order to determine freeze–thaw damage and deterioration mechanism, the initial moisture content of gray brick specimens was set as 20%, 40%, 60%, 80%, 100%. The effects of moisture content and the number of freeze–thaw cycles on the quality, mechanical properties and microstructure of gray brick were investigated by uniaxial compression tests and scanning electron microscopy (SEM) tests. Numerical simulations were applied to model the freezing and thawing process. The results showed that: as the number of freeze–thaw cycles increased, the mass loss rate and peak strength reduction rate of gray brick both increased. The initial moisture content had a greater impact on damage to gray brick due to freeze–thaw; *ω* = 80% was defined as the limit moisture content of gray brick. Under the repeated action of freeze–thaw cycles, the areas affected by thermal stress were mainly concentrated in the center of the outer surface and the center of the side of gray bricks. The maximum thermal stress after 55 freeze–thaw cycles was 1.522 × 10^−2^ MPa. This research results provide a theoretical basis for the prevention and protection of frost damage of brick buildings in a freeze–thaw environment.

## 1. Introduction

Located on a high platform 1.5 km southeast of Kaifeng City, Henan Province, the Po Pagoda was built in the 7th year of Kaibao during the Northern Song Dynasty (974 A.D.). It is a national-level protected cultural relic with a brick-wood structure. Gray brick is an ancient building material made of clay fired at high temperature [1], which has the characteristics of high porosity, loose texture, low strength, strong water absorption and poor frost resistance [2,3] As the gray bricks are exposed to the open environment for a long time, they are vulnerable to the effects of rain, snow, temperature, seasonal freeze–thaw cycles and other climatic conditions, resulting in different degrees of weathering damage on their surfaces, such as pulverization, desquamation and cracks. Among them, the effect of freeze–thaw cycles in reducing the stability of these materials and causing their premature failure cannot be ignored [4,5,6,7]. In addition, the influences of groundwater, seepage and environmental humidity give rise to fluctuations in the moisture content within the bricks, resulting in variations in water content [8,9]. Coupled with the action of frost, the interactions make freeze–thaw damage of gray brick more complicated. Therefore, research on freeze–thaw damage and the deterioration mechanisms of gray bricks with different water contents is important for the prevention of frost damage and the protection of existing brick buildings.

In recent years, researchers have examined the characteristics of bricks under freezing and thawing environments, and have obtained a number of results. Liu and Zhang found that salt crystallization and freeze–thaw cycles were important factors leading to brick weathering through on-site investigations and indoor experiments, and formulated antiweathering strategies [10]. Tang et al. studied the effects of freeze–thaw action and saturation on the mechanical properties of ancient bricks and mortar [11]. Grubeša et al. researched the influence of pore size distribution and pore type and their connection to the frost resistance of bricks by porosity measurements and microcomputed X-ray tomography (micro-CT) [12]. Uranjek et al. found that the ductility of brick masonry deteriorated in a freeze–thaw environment, and built a mathematical statistical model based on experimental data for probabilistic risk assessments [13]. Zhang and coauthors took ancient bricks as their research object, and studied the effects of free-thaw on quality, porosity and compressive strength [3]. The freeze–thaw damage process and mechanisms on ancient gray bricks were also explored with the help of TEM scanning. Grubeša et al. investigated the influence of pore size distribution on the frost resistance of clay bricks on the basis of mercury intrusion porosity measurements and freeze–thaw cycle tests [14]. Scholars have analyzed and summarized the changes in the physical and mechanical properties of bricks under freeze–thaw environments, but most such studies have been based on macro-experimental phenomena, and few studies have been conducted to reveal the mechanism of freeze–thaw damage in gray bricks in depth.

Khanlari et al. took five different types of sandstone as their research object, studying the influence of freeze–thaw cycles on the physical and mechanical properties; they also used the attenuation function model to evaluate the durability of sandstone in a freeze–thaw environment [15]. Hosseini and Khodayari focused on Lushan sandstone and examined the influence of freezing temperatures and the number of freeze–thaw cycles on the deterioration of rock strength [16]. Hagan et al. studied the effect of temperature, loading rate and water content on the strength and failure process of frozen rock [17]. Qiao et al. revealed the evolutionary characteristics of frost heave pressure caused by the water–ice phase transition and volume expansion in fractured rock bodies, and the influence of freeze–thaw cycles on the mechanical properties of fractured rock masses was analyzed [18]. Lv and coauthors selected gneisses in the Taxian region of Xinjiang for freeze–thaw cycling tests to study the changes in the microstructure and macroscopic physical properties of the rock samples, revealing the mechanism of freeze–thaw deterioration and collapse of the rock mass [19]. Rong et al. used a microscope to qualitatively analyze the microstructure of porous yellow sandstone under a freeze–thaw environment [20]. The damage characteristics were also quantitatively investigated using a pore size analyzer, and the weakening mechanism of single-hole yellow sandstone was described in terms of its microstructure. The present research shows that compared with the research results on freeze–thaw damage to rocks, less research has been done on brick freeze–thaw damage, and the deterioration mechanisms therein are not yet well understood.

In view of this, this work takes the protection of existing brick buildings such as the Po Pagoda as its research background, and uses gray bricks with different moisture content as the research object to conduct freeze–thaw cycle tests. The effects of moisture content of gray brick and the effects of freeze–thaw cycles on their compressive strength were evaluated through uniaxial compression tests. Based on SEM images, the influence of the number of cycles on the microstructure are analyzed. In addition, numerical simulations are used to build temperature field and thermal stress field models of a gray brick specimen with which to simulate freeze–thaw cycles and further reveal the damage and deterioration mechanisms that these cause in gray brick.

## 2. Materials and Methods

### 2.1. Materials and Preparation of Specimens

#### 2.1.1. Material Properties

The brick used in this experiment came from Kaifeng City, Henan Province. It was a gray brick which had been discarded following the demolition of old residential buildings, and was steel-gray in color. Through using an X-ray diffractometer (Rigaku D/max 2400; Rigaku, Japan), its main mineral components were found to be quartz, anorthite, albite and microplagioclase. The chemical compositions of gray brick were obtained using X-ray fluorescence spectrometer (Rigaku ZSX Primus; Rigaku, Japan); the results are shown in Table 1.

#### 2.1.2. Preparation of Specimens

In order to reduce the influence on the results of the variability of the test materials, bricks with similar natural moisture contents were first selected, and then 36 test pieces with a size of 50 mm × 50 mm × 50 mm were divided by a cutting machine. With six specimens in each group, the 36 bricks were divided into six groups according to their water contents; each sample was then numbered. The first Arabic numeral represents water content (water contents in this experiment were classified as 20%, 40%, 60%, 80% and 100%), while the second Arabic numeral indicates the serial number of the one group. The first three specimens of each group were tested in freeze–thaw cycles alone, while the last three were subjected to uniaxial compression tests before undergoing freeze–thaw cycles.

After cleaning the surfaces of the samples with a brush, they were placed in a blast drying oven at a temperature of 105 °C and dried to a constant weight (during the drying process, they were weighed every 30 min, and the difference in mass between two measurements was not more than 0.2%). The specimens were removed and cooled to room temperature, and then weighed to obtain the dried mass $m_{d}$. These specimens were soaked in clean water for 24 h and then weighed to obtain the saturated mass $m_{s}$. The corresponding mass of the samples with different moisture contents $m_{t}$ were obtained according to Equation (1). The saturated specimens were left to dry naturally, and were then weighed at regular intervals until the desired moisture content had been reached. Specimens were sealed with plastic wrap and then packed in plastic bags to ensure a constant moisture content before freeze–thaw cycles tests [21,22,23].

(1) $$m_{t}=\;\left(1-\omega \right)m_{d}{\;+\;\omega m}_{s}$$

where $m_{t}$ is the corresponding mass of the specimens with different moisture contents (kg); $m_{d}$, $m_{s}$ is the mass of dried and saturated specimens (kg), respectively; and ω is the water content of the specimen (%).

### 2.2. Experiment Methods

#### 2.2.1. Freeze–Thaw Test

**Figure 1:**
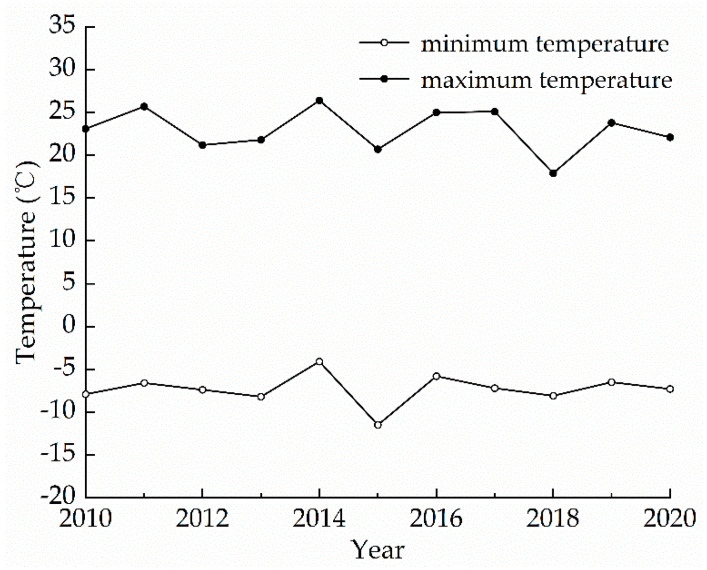
The minimum and maximum temperatures in winter in Kaifeng from 2010 to 2020.

The minimum and maximum temperatures in winter in Kaifeng from 2010 to 2020 are shown in Figure 1. By referring to this, −12 °C was selected as the freezing temperature and 26.5 °C as the melting temperature.

**Figure 2:**
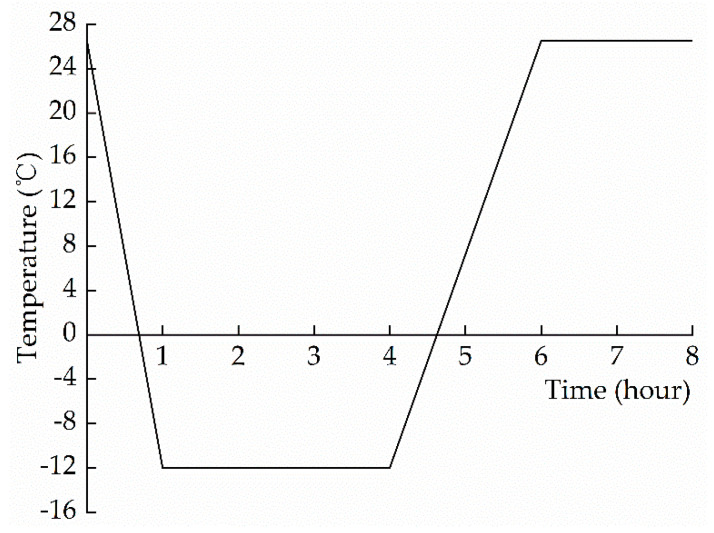
Temperature change during one freeze–thaw cycle.

Before the start of freeze–thaw cycles, the samples with different water contents were sealed for 7 days to keep the moisture in them uniformly distributed. According to the relevant specifications and requirements [24], the samples were taken out and wiped with a damp cloth, before being placed in a sealed transparent box at a separation distance greater than 20 mm. The plastic box was then placed in a constant temperature and humidity chamber at a temperature of 26.5 °C and a relative humidity of 55% RH. When the temperature inside the chamber changed to 26.5 °C, a timer was started. It took 1 h to cool to −12 °C. The temperature was maintained at −12 °C for 3 h. Then, it took 2 h to heat to 26.5 °C; the chamber was maintained at this temperature for 2 h. This is counted as one freeze–thaw cycle; the temperature change during one freeze–thaw is shown in Figure 2. This cycle was repeated 55 times for each group of samples. After every five cycles, the samples were taken out, their external appearance was checked and recorded, and the quality of each specimen was measured.

#### 2.2.2. Uniaxial Compression Test

After the 55 cycles were over, the uniaxial compression test was carried out on all specimens at a loading rate of 1 mm/min using a WDW-600 microcomputer-controlled electronic universal testing machine. The mechanical parameters of the specimens with different moisture contents before and after freezing and thawing, such as peak strength and peak strain, were obtained.

#### 2.2.3. Scanning Electron Microscope Test

At the end of each five cycles, according to the compression failure characteristics of each group of samples, sections of typical parts were selected to make slices for electron microscopy scanning.

## 3. Experimental Results

### 3.1. Appearance Change

**Figure 3:**
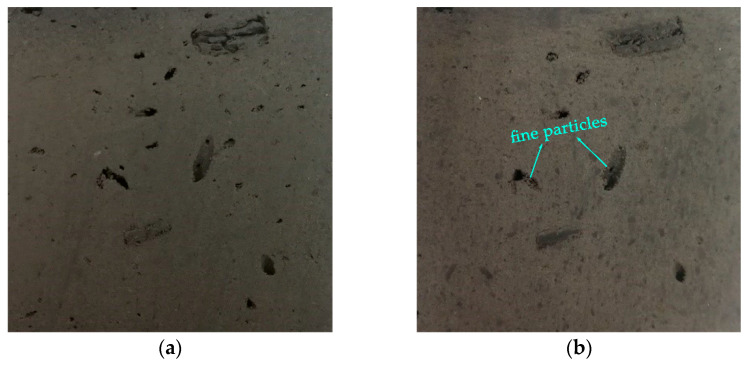
Images of the failure characteristics of gray brick specimens before and after freezing and thawing cycle: (**a**) the 0th freeze–thaw cycle; (**b**) the 55th freeze–thaw cycle.

Deterioration of the outer surface of the gray brick specimens was observed and recorded at the end of every five cycles. Figure 3 demonstrates the failure characteristics before and after freezing and thawing cycles. Comparing the specimens, it was evident that the surfaces of gray bricks which had undergone freeze–thaw cycles were different from those that had not, with the former becoming obviously rougher to the touch. Observed from the side of gray brick samples, their surface was no longer relatively smooth, and there were loose fine particles. After touching by hand, there was a sandy feel and the phenomenon of small particles falling off. This was because water molecules in the pores and cracks of gray brick had the effect of softening the mineral skeleton, so the cement between particles underwent dissolution and dissolution reactions [25,26,27]. At the same time, minerals such as albite and anorthite in gray brick reacted with water to produce various secondary minerals, resulting in interparticle cements which gradually became detached [28]. However, in contrast to the research results of others [29], there were no obvious cracks, delaminations or bulges on the surface of the gray brick after the end of freeze–thaw cycles, which is related to the difference in temperature range and the number of freeze–thaw cycles.

### 3.2. Quality Change

The gray brick specimens were weighed after each five freeze–thaw cycles. The mass loss rate of the specimens was calculated according to Equation (2), and the average value under the same moisture content was found.

(2) $$M=\frac{m_{0}-m_{N}}{m_{0}}\;\times \;100\%\;$$

where M is the mass loss rate of the gray brick specimens (%); $m_{N}$ is the mass of the specimens after N freeze–thaw cycles (kg); and $m_{0}$ is the initial mass of the specimens (kg).

**Figure 4:**
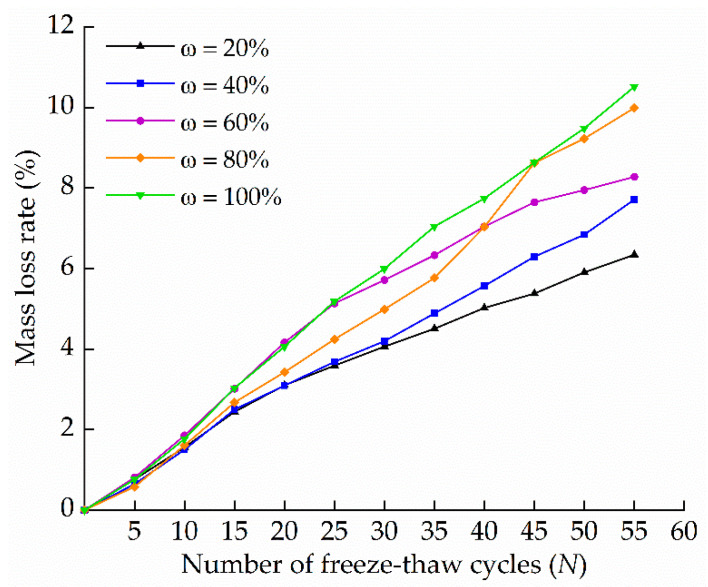
Relationship between mass loss rate and the number of freeze–thaw cycles of gray brick samples with different moisture contents.

Figure 4 shows the relationship between mass loss rate and the number of freeze–thaw cycles of gray brick samples with different moisture contents. The results showed that with the increase of the number of freeze–thaw cycles, the mass loss rate of gray brick samples with different moisture contents gradually increased. In the freeze–thaw cycle, the water in the gray brick undergoes water phase changes, so the gray brick particles suffer from fatigue damage and the bonding force between particles decreases, resulting in the arrangement of particles becoming looser. With an increase in the number of cycles, the flaking phenomenon of gray brick particles became more and more visible, which is indicative of a decline of quality.

When the number of freeze–thaw cycles was the same, the mass loss rate increased with an increase of moisture content. After completing 55 cycles, the mass loss rates of the specimens with moisture contents of 20%, 40%, 60%, 80% and 100% were 6.34%, 7.71%, 8.28%, 9.99% and 10.52%, respectively. This was due to the frost swelling force generated when water was converted into ice in a low temperature environment. When the frost swelling force was greater than the cementing force between mineral crystals, it led to structural deterioration; the lower the moisture content of gray brick, the smaller the frost swelling force produced by water [30]. Therefore, after the same number of freeze–thaw cycles, the specimen with a water content of 20% was weakly affected and the decline in quality was not large. In contrast, the sample with a water content of 100% suffered from the greatest degree of freeze–thaw damage, and the change in quality was the most obvious. From these results, it is clear that a greater moisture content in gray brick results in more obvious damage due to freezing–thawing.

### 3.3. Mechanical Properties

In terms of the relevant specifications and requirements [24,31], the specimens with different moisture contents were selected for uniaxial compression tests before and after freezing and thawing. The appearance changes of the gray brick specimens were observed during the test, and their mechanical properties were obtained.

#### 3.3.1. Damage Characteristics of Uniaxial Compression

**Figure 5:**
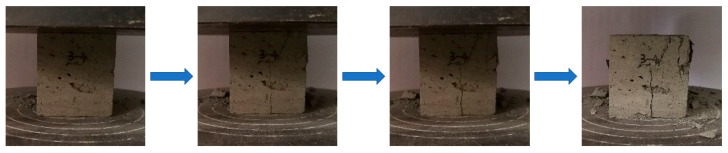
Change in the appearance of gray brick specimen during uniaxial compression.

Taking the gray brick sample with a moisture content of 40% as an example, the compression failure characteristics under a freeze–thaw environment were summarized. Figure 5 shows the change in the appearance of gray brick specimen during uniaxial compression (the corresponding loads for each photo from left to right are 2.45 kN, 7.23 kN, 9.29 kN and 12.90 kN, respectively). It can be seen that the failure mechanisms under uniaxial compression condition were as follows: the surface of the specimen first gradually formed a crack through the top and bottom, and multiple cracks appeared over time. The fracture plane gradually expanded from a single to composite, multiple fracture planes. Rupture and sample block spalling occurred on the surface of the specimen, and mostly showed flaky peeling. This indicated that gray bricks exhibit obvious brittle failure characteristics after freeze–thaw cycles.

From the uniaxial compression failure forms of gray brick after freezing and thawing, it can be seen that the gray brick was damaged and destroyed; its strength reduces under a freeze–thaw environment, leading to multiple cracks on its surface under the action of external force. With the continuous application of load, the cracks gradually expanded and penetrated, resulting in obvious brittle damage characteristics. As a result, the gray brick presented flaky peeling and compound multifracture surfaces under uniaxial compression condition.

#### 3.3.2. Peak Strength

The peak strength of all gray brick samples before and after freeze and thawing was evaluated, and the peak strength reduction rate was calculated according to Equation (3).

(3) $$F=\frac{f_{55}-f_{0}}{f_{0}}\;\times \;100\%$$

where F is the peak strength reduction rate of the gray brick samples (%); $f_{55}$ is the peak strength of the specimens at the end of the freeze–thaw cycles (MPa); and $f_{0}$ is the peak strength of the specimens before undergoing freeze–thaw cycles (MPa).

Table 2 shows the peak strength of the gray brick specimens with different moisture contents before and after freeze–thaw cycles. The overall change laws of peak strength of the specimens are summarized from Table 2. When fifty-five cycles had been completed, the peak strength of all gray bricks had decreased. The peak strength reduction rates of the specimens with different moisture contents (from small to large) were 18.22%, 23.38%, 23.84%, 26.13% and 30.83%, respectively, showing an increasing trend with the increase of moisture content. The gray brick with a water content of 20% had the lowest peak strength reduction rate, while the saturated group had the highest. This analysis suggests that under the repeated action of freezing and melting, the water in brick undergoes phase changes. At the same time, various minerals also shrink and expand at different rates, leading to a constant change in volume. These changes cause microcracks to occur. With the increase in the number of freeze–thaw cycles, the cracks continued to expand and penetrate, causing freeze–thaw damage. This led to irreversible damage to the mechanical properties of the material. Consequently, the peak strength of the gray brick samples was reduced.

### 3.4. Microstructure Characteristics

**Figure 6:**
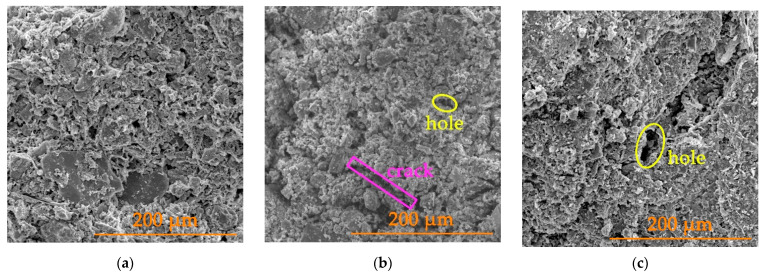
SEM images of a gray brick with a moisture content of 40% under different cycles: (**a**) the 0th freeze–thaw cycle; (**b**) the 30th freeze–thaw cycle; (**c**) the 55th freeze–thaw cycle.

**Figure 7:**
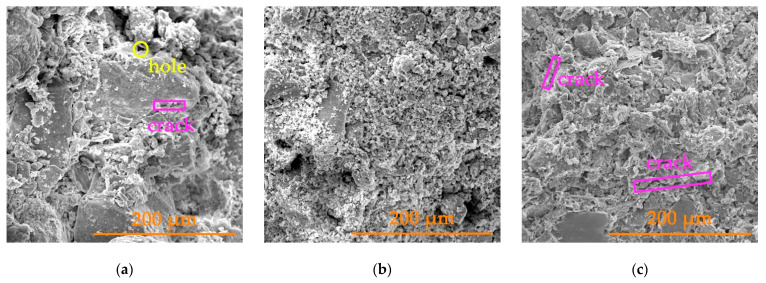
SEM images of a gray brick with a moisture content of 100% under different cycles: (**a**) the 0th freeze–thaw cycle; (**b**) the 30th freeze–thaw cycle; (**c**) the 55th freeze–thaw cycle.

In order to more intuitively observe the microstructural characteristics of the gray brick with different water contents in a freeze–thaw environment, SEM images were obtained by scanning electron microscopy using 500 times magnification for the specimens that had undergone different numbers of freeze–thaw cycles. Figure 6 and Figure 7 show SEM images of the gray brick with moisture contents of 40% and 100% under different cycles, respectively.

From the results in Figure 6 and Figure 7, it can be seen that the gray brick has a porous structure with varying pore sizes. The internal particle size of the gray brick prior to freeze–thaw cycle is large; the particles have obvious angular characteristics and the edges of the particles are clear. After 30 to 55 cycles, the brick particles decreased with an increase in the number of cycles, and the large particles were gradually broken down into small particles. The edge lines of the particles became rounded and no longer had distinct angular features. These phenomena indicate that under the action of repeated freezing and thawing, the gray bricks are continuously subjected to frost heave and unloading [3,32], having the effect of smoothing effect on the edges after repeated grinding.

With the repeated action of freezing and thawing, the original pores in brick continued to expand, and the cracks kept developing. The main manifestations were that the diameters of the pores increased continuously, and the length and width of the cracks also increase. Our analysis concluded that there were a large number of pores inside the gray brick which can absorb and transfer liquid water. When temperature drops, liquid water gradually condenses into ice. This phase change expands the volume of water and forms an expansion pressure which resists the restriction of the capillary wall. In contrast, the gray brick bears tensile stress. When the temperature rises, the ice gradually melts into liquid water, resulting in irreversible expansion and deformation. At the same time, the minerals in the brick react with water to generate secondary minerals. The decomposition of old materials and the generation of new substances changes the original pore structure [2,33]. With more freeze–thaw cycles, the microstructure of brick becomes progressively more damaged. This damage accumulates and eventually manifests as a reduction in the macroscopic mechanical properties of the brick. Macroscopic destruction of gray brick is closely related to microscopic structural deterioration.

## 4. Numerical Simulation of Freeze–Thaw Damage to Gray Bricks

Analyses only based on macroscopic tests have a certain degree of one-sidedness and cannot show the distribution patterns and change of temperature and thermal stress during the freezing and thawing process. Therefore, on the basis of our freeze–thaw cycle tests, the numerical simulation software ABAQUS/CAE 2016 was used to establish a finite element model of the gray brick specimen. The temperature and thermal stress distribution and evolution laws of the specimen during the freezing and thawing process were analyzed, and the freeze–thaw deterioration mechanism in gray brick was further revealed.

### 4.1. Build Models

This paper focuses on the damage and deterioration of gray brick under the action of freeze–thaw cycles. Under the influence of temperature, the thermal stress generated is the direct cause of freeze–thaw damage, while the presence of stress and strain does not affect temperature. Therefore, the sequential coupled thermal stress analysis method was adopted, that is, the temperature field simulation was performed first and the temperature field simulation result was imported into the thermal stress field as a predefined field for thermal stress analysis [34,35].

#### 4.1.1. Model of Temperature Field

A cube model with a size of 50 mm × 50 mm× 50 mm was established, and the temperature field was simulated and analyzed. We created a heat transfer analysis step. As part of the interaction module, we created both thermal radiation and thermal convection interactions. We also created a temperature predefined field under the load module and set the temperature to 25 °C to restore the gray brick to its initial temperature state. We assigned the element type and set to heat transfer. In order to facilitate the comparison with the macroscopic test results, the amplitude of temperature was edited according to the curve shown in Figure 2 to realize the simulation of the freezing and thawing process. The temperature range was from −12 °C to 26.5 °C, the period of one freeze–thaw cycle was eight hours, the number of cycles was 10, 20, 30, 40, 50 and 55, and the corresponding times were 2.88 × 10^5^ s, 5.76 × 10^5^ s, 8.64 × 10^5^ s, 1.152 × 10^6^ s, 1.44 × 10^6^ s and 1.584 × 10^6^ s. The parameters of the gray brick were selected according to the specification requirements [31,36] and related documents, [37,38], as shown in Table 3.

#### 4.1.2. Model of Thermal Stress Field

When performing our thermal stress analysis, the established model and divided grid density were consistent with the temperature field. The element type was assigned as 3D stress. This step was set to static-general. At the same time, the simulation result of the temperature field was imported into the thermal stress analysis model as a predefined field.

### 4.2. Simulation Results

#### 4.2.1. Simulation Results of Temperature Field

**Figure 8:**
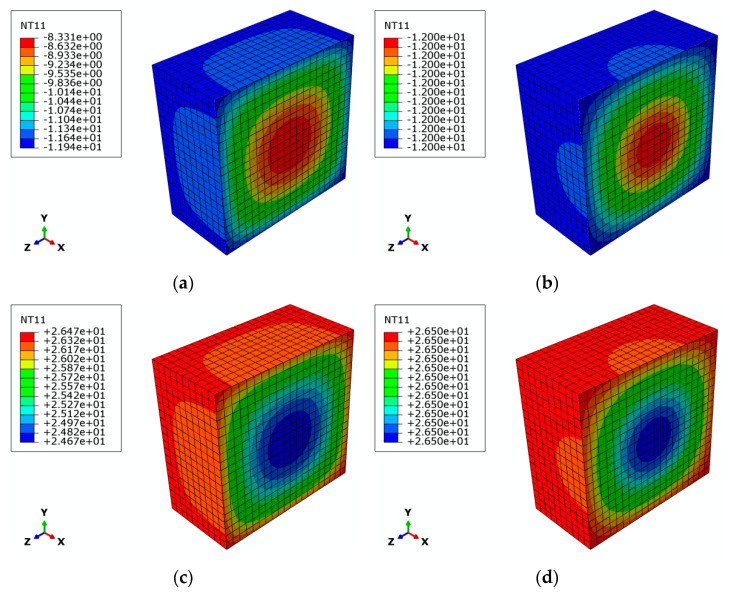
Distribution state and overall change process of the temperature of gray brick from inside to outside during one freeze–thaw cycle: (**a**) the 1st hour; (**b**) the 1.7th hour; (**c**) the 6th hour; (**d**) the 6.7th hour.

The distribution state and overall change of temperature of the model from inside to outside during one freeze–thaw cycle are shown in Figure 8. After conducting freeze–thaw cycles for 1 h, the temperature of model was distributed in a circular pattern, increasing layer by layer from the outside to the inside. During this period, the lowest temperature of the outer layer dropped from 25 °C in its initial state to −19.4 °C, while the highest internal temperature was −8.331 °C. When the freeze–thaw cycles reached 1.7 h, the temperature of the entire model dropped to −12 °C, where it remained until the 4th hour. After the freeze–thaw cycles had been carried out for 6 h, the temperature still showed a circular distribution, but the temperature decreased from the outside to the inside, layer by layer. Finally, the maximum temperature of the outer layer increased from −12 °C at the end of four hours to 26.47 °C. The minimum internal temperature was 24.67 °C. When the freeze–thaw cycles had been carried out for 6.7 h, the temperature of the whole model increased to 26.5 °C, where it remained until the 8th hour (end of one cycle). During the 55 cycles of freezing and thawing, the temperature changed cyclically.

The state and change of temperature indicate that during the freeze–thaw cycles, the temperature of the gray brick showed a circular distribution which changed layer by layer from exterior to interior. In the process of lowing the temperature, the external temperature was lower than the internal temperature. During the heating process, its external temperature was higher than the internal. Thus, temperature differences between inside and outside occurred. After experiencing many freeze–thaw effects, the continuous change of internal and external temperature caused the gray brick to be significantly affected by thermal stress, resulting in freeze–thaw damage. The simulation results of the temperature field also show that the internal temperature change of the gray brick always lagged behind the external temperature. This allowed the outer temperature to remain stable for a longer time compared to the inner one. Accordingly, the outer layer was affected by high or low temperatures for a longer time, and thus, the outer layer suffered more severe freeze–thaw damage than the inner layer.

#### 4.2.2. Simulation Results of Thermal Stress Field

**Figure 9:**
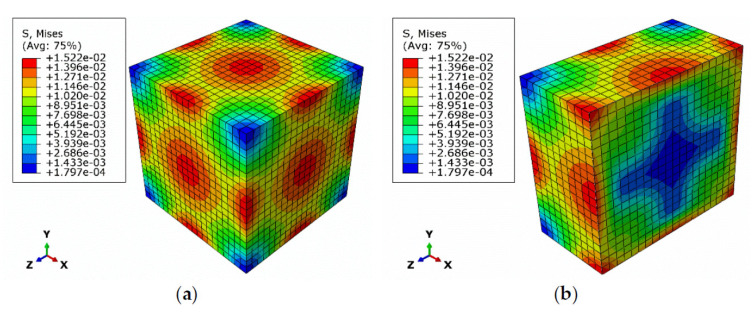
Thermal stress cloud in the last freeze–thaw cycle: (**a**) outer surface of the model; (**b**) profile of the model.

Under the repeated actions of freezing and melting, heat is gradually transferred from the outer layer of the model to the inside. This causes the temperature change of the inner layer to lag behind that of the outer one, and a temperature difference between the two occurs which generates thermal stress and strain. Figure 9 shows the thermal stress cloud during the last freeze–thaw cycle. It can be seen that after the freeze–thaw simulation had been completed, the maximum thermal stress on the gray brick model was 1.522 × 10^−2^ MPa. The areas that were most affected were mainly located in the surface center and the center of the edges, while the less affected regions were mainly concentrated in the corners and the inner center.

At the end of the freeze–thaw test, the part of gray brick that was most affected by thermal stress was in the outer layer. The maximum depth of influence was about 2.5 mm, which accounts for one-tenth of the thickness of the specimen. It can be seen that following long-term freeze–thaw influence, not only the surface, but also the internal part at a certain depth of the of gray brick will be damaged.

## 5. Conclusions

In this study, freezing and thawing test were carried out on gray bricks with different moisture contents. The effects of water content and freeze–thaw action on the peak strength of the gray brick were determined by uniaxial compression tests. The effects of the number of cycles on their microstructure were analyzed based on SEM images. The temperature field and thermal stress field models were constructed by numerical simulation to simulate the freeze–thaw cycling process. By analyzing the research results, the following conclusions may be drawn:

1. The freeze–thaw action has an impact on the macroscopic properties of gray brick. As the number of freeze–thaw cycles increases, the quality and peak strength show a downward trend. After 55 freeze–thaw cycles, the mass loss rate of gray brick with different moisture contents is greater than 6%, and the maximum peak strength reduction rate exceeds 30%.
2. After 55 cycles under the same freeze–thaw conditions, when the initial moisture content is ≥80%, the mass loss rate and peak strength reduction rate of the sample are significantly greater than those below 80%. It can therefore be concluded that the initial moisture content has the greatest impact on the freeze–thaw damage of gray brick, and as such, *ω* = 80% may be defined as the upper limit of the moisture content. When the moisture content of gray brick is less than 80%, its mechanical properties show more obvious deterioration.
3. Freeze–thaw action makes the edges of the gray brick particles rounded and removes their obvious angular features. With the increase in the number of freeze–thaw cycles, the pore diameter inside the gray brick increases, and cracks gradually develop.
4. With more and more freeze–thaw cycles, the temperature of gray brick presents a circular distribution and changes layer by layer. The temperature changes inside the specimen always lag behind those on the surface, i.e., the temperature on the surface remains stable for longer than inside the brick. With an increase in the number of freeze–thaw cycles, the maximum thermal stress on the specimen model gradually increases, and the damage becomes more and more pronounced. Moreover, the areas severely affected by thermal stress are mainly concentrated on the outer surface center and side center of the test block model. The corners and inner center areas are less affected by thermal stress.

## Figures and Tables

**Table 1:** Chemical compositions of gray brick.

Compound	SiO_2_	Al_2_O_3_	CaO	Fe_2_O_3_	MgO	K_2_O	Na_2_O	TiO_2_
Concentration (%)	59.290	17.630	8.690	5.230	3.200	2.740	1.950	0.683

**Table 2:** Peak strength of the gray brick specimens with different moisture contents before and after freeze–thaw cycles.

Moisture Content (%)	Peak Strength (MPa)		Strength Reduction Rate (%)
	Before Freeze–Thaw	After Freeze–Thaw	
20	7.41	6.06	18.22
40	5.86	4.49	23.38
60	2.81	2.14	23.84
80	8.84	6.53	26.13
100	6.13	4.24	30.83

**Table 3:** Parameters of the gray brick.

Density (kg/m^3^)	Elastic Modulus (MPa)	Poisson’s Ratio	Coefficient of Thermal Expansion (°C^−1^)	Thermal Conductivity (W·m^−1^·K^−1^)	Specific Heat Capacity (J·kg^−1^·K^−1^)
1635	1807	0.15	5 × 10^−6^	0.81	1050

## Data Availability

The data presented in this study are available on request from the corresponding author.
